# Force Transmission by Minimal Focal Adhesion Complexes Induces Synthetic Cell Deformation

**DOI:** 10.1021/acssynbio.5c00645

**Published:** 2025-12-17

**Authors:** Natalie Huhn, Chiao-Peng Hsu, Timon Nast-Kolb, Arsenii Hordeichyk, Andreas R. Bausch

**Affiliations:** † Heinz Nixdorf Chair in Biophysical Engineering of Living Matter, Center for Functional Protein Assemblies, Center for Organoid Systems, Department of Bioscience, 9184 Technical University of Munich, Technical University of Munich School of Natural Sciences, Garching 85748, Germany; ‡ Department of Physics, Friedrich-Alexander-Universität Erlangen-Nürnberg, Erlangen 91058, Germany; § Max Planck School Matter to Life, Heidelberg 69120, Germany; ∥ Clinic for Children and Adolescent Medicine, Friedrich Alexander University Erlangen-Nuremberg, Erlangen 91054, Germany

**Keywords:** reconstitution, focal adhesion, actin, actomyosin, protein complex, contractile force transmission

## Abstract

Cells sense and respond to mechanical cues through focal adhesions–dynamic, multiprotein assemblies linking the actin cytoskeleton to the extracellular matrix. These complexes are essential to processes from cell migration to tissue morphogenesis, yet the minimal physical requirements for their force-transmitting and mechanosensing functions remain unclear. Here, we reconstitute minimal focal adhesion-like complexes in giant unilamellar vesicles (GUVs) using kindlin-2, talin-1, FAK, paxillin, zyxin, and VASP anchored to membranes containing PIP_2_ and integrin β1 tails. These assemblies nucleate and anchor actin filaments into networks spanning the vesicle surface. Upon addition of nonmuscle myosin IIa, actomyosin contraction thickens filament bundles, aligns the complexes, and deforms the GUVs, while the assemblies remain stably membrane-bound. Our findings show that actin recruitment, force transmission, and structural stability under load can emerge from defined protein-membrane interactions alone. This minimal, three-dimensional platform offers a controllable synthetic biology system for probing mechanosensing and engineering force-responsive biomimetic systems.

## Graphical abstract

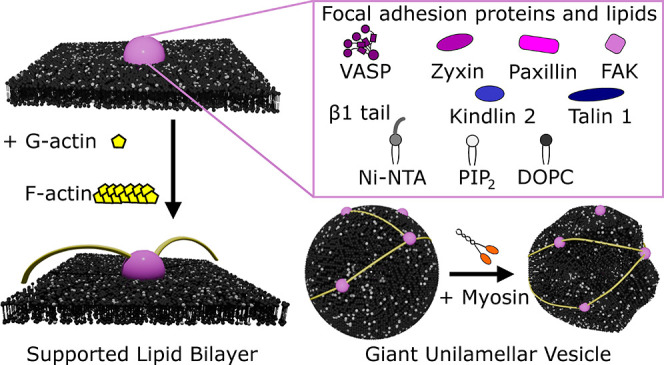

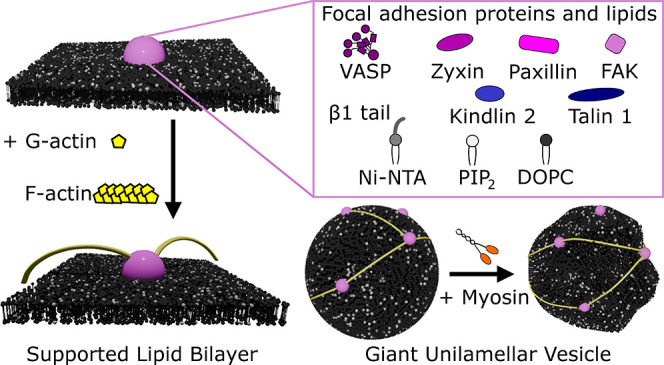

## Introduction

Synthetic cells are minimal model systems designed to reproduce key cellular functions in a controlled and simplified format. Rather than capturing the full complexity of living cells, they typically reconstitute specific subsystems, enabling precise dissection of individual biological processes.^1,2^ These minimal models provide a valuable platform to study complex biological phenomena in a simplified setting.^3,4^ Such bottom-up approaches have provided valuable insights into cytoskeletal organization, signaling, and membrane mechanics.^5^ One widely studied subsystem is the actin cortex, where reconstituted actin filaments and associated proteins have been shown to display dynamic behaviors such as wave propagation and symmetry breaking, recapitulating cortical patterns observed in vivo.^6^ These systems have revealed feedback mechanisms that organize the cortex and regulate cell shape. Encapsulation of actomyosin networks in lipid vesicles, for example, has reproduced essential aspects of cellular mechanics and advanced our understanding of active systems.^7–10^ Adhesion interactions represent another key focus of synthetic cell studies, offering a means to explore how cells attach, transmit forces, and sense their environment.^11,12^

Building on models of the actin cortex and adhesion, synthetic systems have begun to explore focal adhesion (FA) proteins and their interactions with actin networks to better understand their cellular roles.^13,14^ Most approaches connect the active components to model membranes through biotin-streptavidin or biotin-neutravidin coupling.^7,9,10,15,16^ While these methods enable actin-membrane linkage, they do not replicate the direct interactions between the membrane, bridging proteins, and actin filaments found in cells.^17^ As a result, the force-bearing and organizational functions of membrane-bound FA proteins in three-dimensional contexts remain largely untested. Focal adhesions are multiprotein assemblies that link the extracellular matrix to the cytoskeleton via integrins and play essential roles in cell migration, adhesion, mechanosensing, and cytoskeletal organization.^18–23^ Understanding their minimal functional requirements is challenging in vivo due to their small size, rapid turnover, and complex regulation.^24^ Minimal synthetic systems offer a route to isolate and probe these functions under defined conditions. Focal adhesions form through dynamic protein–protein and protein–lipid interactions, with integrins providing the transmembrane link to the extracellular matrix. Inside the cell, integrin activation is mediated by proteins such as talin and kindlin, which bind to the β-integrin cytoplasmic tail and to phosphatidylinositol 4,5-bisphosphate (PIP_2_), stabilizing the active conformation.^24,25^ Activated integrins recruit additional adaptor and signaling proteins, including focal adhesion kinase (FAK), paxillin, and zyxin, which in turn engage actin-binding proteins such as vasodilator-stimulated phosphoprotein (VASP).^26–28^ This assembly provides multiple actin attachment sites, enabling the integration of actin filament growth with adhesion maturation. Recent studies have shown that many focal adhesion components undergo liquid–liquid phase separation (LLPS), both in vivo and on model membranes,^29–33^ suggesting that condensate formation may facilitate the rapid assembly, disassembly, and force responsiveness of these structures. However, whether such membrane-bound condensates alone are sufficient to transmit forces and resist mechanical load remains unknown.

Here, we present a minimal, three-dimensional synthetic cell system to test the force-bearing function of reconstituted focal adhesion-like complexes. Using giant unilamellar vesicles (GUVs) functionalized with PIP_2_ and integrin β1 tails, we sequentially assemble six key focal adhesion proteins–kindlin-2, talin-1, FAK, paxillin, VASP, and zyxin–into membrane-associated condensates. These complexes nucleate and anchor actin filaments into networks spanning the vesicle surface. Addition of myosin II generates contractile forces that thicken actin bundles, align complexes, and deform the GUVs, while maintaining membrane anchorage. This minimal, three-dimensional system isolates the physical principles of force transmission and provides a versatile platform for engineering force-responsive biomimetic structures.

## Results

### Focal Adhesion-like Complex Formation on Supported Lipid Bilayers

We first reconstitute membrane-associated focal adhesion-like complexes on supported lipid bilayers (SLBs) as a two-dimensional model system. The SLBs contain phosphatidylinositol 4,5-bisphosphate (PIP_2_) and integrin β1 tails attached to Ni-NTA lipid via a His-tag. In sequential incubation steps, we add kindlin-2, talin-1, focal adhesion kinase (FAK), paxillin, zyxin, and vasodilator-stimulated phosphoprotein (VASP) to the model membranes (Materials and Methods, Figure 1a). These proteins interact with each other and with PIP_2_, driving condensate formation on the membrane.^30^

**Figure 1:**
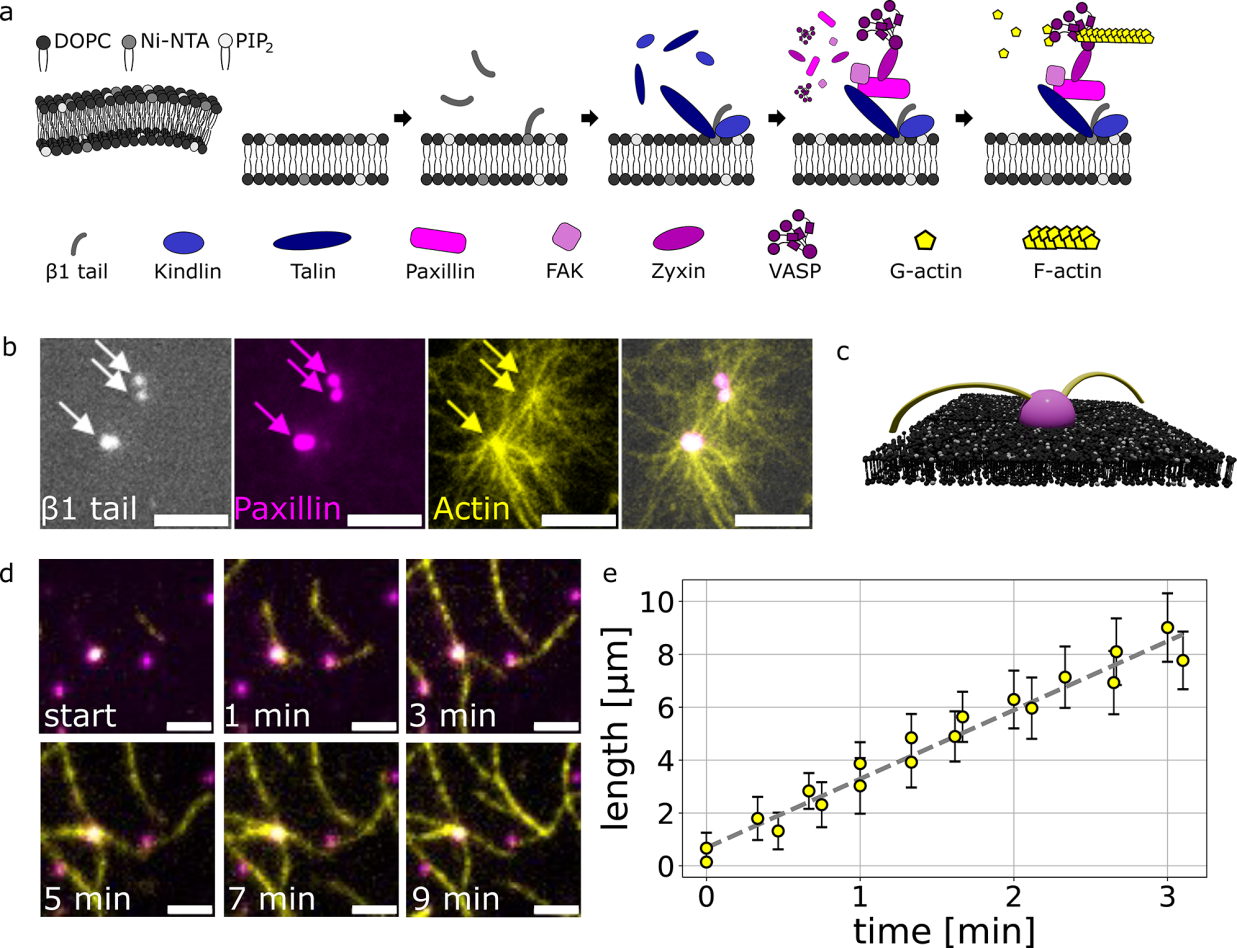
The growth of actin filaments from focal adhesion protein complexes on SLBs. Actin filaments grow from focal adhesion protein complexes on SLBs, connecting the complexes with an actin network. The complexes anchor the actin filaments toward the lipid membrane. (a) Schematic of the reconstituted model membranes and the incubation steps driving the complex formation and allowing for actin binding and polymerization. (b) Actin filament network (yellow, total internal reflection fluorescence) links to the β1 clusters (greyscale, epifluorescence) and the protein condensates (paxillin, magenta, epifluorescence). Scalebars are 5 μm. (c) Schematic of actin filaments connecting the complexes on the SLB. (d) Actin filaments (yellow, total internal reflection fluorescence) growing and elongating from the focal adhesion protein complexes (paxillin, magenta, epifluorescence). Scalebars are 2 μm. (e) Actin filament growth from protein complexes on SLB over time. The tracked filaments show a polymerization rate of 2.6 μm/min. Error bars represent standard deviations of 21 measurements.

Fluorescence imaging shows colocalization of β1 tails and focal adhesion proteins (Figure 1b), indicating that membrane-bound integrin β1 tails are recruited by kindin-2 and talin-1 across the membrane. Although this colocalization is not strictly required for condensate formation, it is influenced by additional protein–lipid and protein–protein interactions.^29,30^ Actin polymerization is initiated by introducing G-actin in a polymerization buffer after the formation of condensates (Materials and Methods), enabling elongation from VASP-rich complexes (Figure 1a–c). The actin filaments grow at a mean rate of 2.6 μm/min, connecting protein complexes into an extended actin network across the SLB (Figure 1d,e and Movie S1). This anchored network defines the spatial architecture of the actin filaments, with multiple filaments emanating from individual complexes and connecting at additional condensates.

### Actin Network Formation Connects the Protein Complexes and Stabilizes the Membrane

We next examine how actin network formation influences membrane properties. Fluorescence microscopy reveals a dense network of filaments linking protein condensates across the bilayer (Figure 2a). To quantify changes in membrane mobility, we perform fluorescence recovery after photobleaching (FRAP) on β1 tails at successive stages of protein assembly (Materials and Methods).

**Figure 2:**
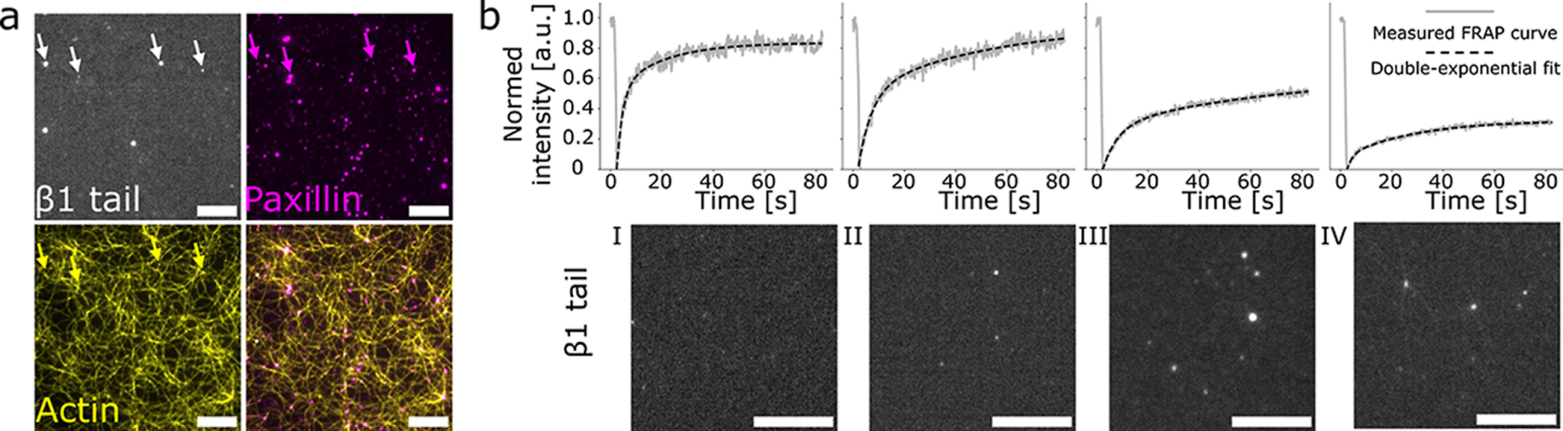
Changes of membrane properties. Membrane properties change with the stepwise introduction of the focal adhesion-related proteins. The fluidity of the membrane decreases after protein complex formation and the actin filament growth. (a) Integrin β1 tails (greyscale, top left, epifluorescence) colocalize with the focal adhesion protein complexes (magenta, top right, epifluorescence) that include fluorescently labeled paxillin on the SLB. Dense network of actin filaments linking protein condensates across the bilayer (yellow, bottom left, total internal reflection fluorescence). Scale bars are 5 μm. (b) FRAP measurements of the fluorescently labeled integrin β1 tail (greyscale, epifluorescence), before (I), after complex formation (II & III), and after the actin network formation (IV). (I) Integrin β1 tail is incubated on a 5% PIP_2_ SLB. The SLB recovers fast and almost fully after photo bleach. (II) 0.5 μM Kindlin-2 and 0.5 μM talin-1 incubated on the membrane change the recovery rate and mobile fraction after bleaching. (III) The incubation of 0.2 μM paxillin, 0.1 μM FAK, 0.2 μM zyxin, and 0.2 μM VASP further changes the recovery curve after the photo bleach. (IV) After actin has polymerized to a network on the SLB, the recovery rate and mobile fraction decrease further. Dotted curves show the double-component exponential recovery fittings. Scale bars are 10 μm.

Compared to membranes containing β1 tails alone, the addition of kindlin-2 and talin-1 reduces the recovery rate, indicating the decrease of β1 tail diffusivity, while the mobile fraction remains similar. Subsequent recruitment of FAK, paxillin, zyxin, and VASP further decreases β1 tail diffusivity, with the mobile fraction dropping from 0.92 to 0.67. Following actin polymerization, mobility is reduced even more, with a mobile fraction of 0.47 (Figure 2b). The mobile fraction of paxillin is also reduced after actin polymerization (Figure S1). These results indicate that the actin network, together with protein condensates, mechanically stabilizes the membrane and restricts lateral diffusion—effects that are expected to also occur in the three-dimensional system. This prompts us to test the properties of the anchoring complexes and the connected actin network on the vesicles regarding their functionality.

### Actin Polymerizes from Focal Adhesion-like Complexes on Giant Unilamellar Vesicles

To extend our observations to a three-dimensional synthetic cell context, we reconstitute focal adhesion-like complexes on giant unilamellar vesicles (GUVs) (Materials and Methods). GUVs produced by electroswelling lead to a uniform PIP_2_ distribution of the membranes (Figure S2). Using the same sequential incubation steps as for SLBs, we assemble kindlin-2, talin-1, FAK, paxillin, zyxin, and VASP on membranes containing PIP_2_ and integrin β1 tails (Figure 3a). The uniformly distributed PIP_2_ promotes the attachment of kindlin-2 and talin-1 on the GUVs’ membrane before the addition of other proteins (Figure S3). Dye influx assays confirm that protein complex formation on the GUV membrane does not alter the membrane permeability (Figure S4). G-actin in a polymerization buffer is added after the formation of condensates (Figure 3a). Fluorescence imaging shows discrete condensates on the vesicle surface that colocalize with β1 clusters and actin polymerization sites (Figure 3b).

**Figure 3:**
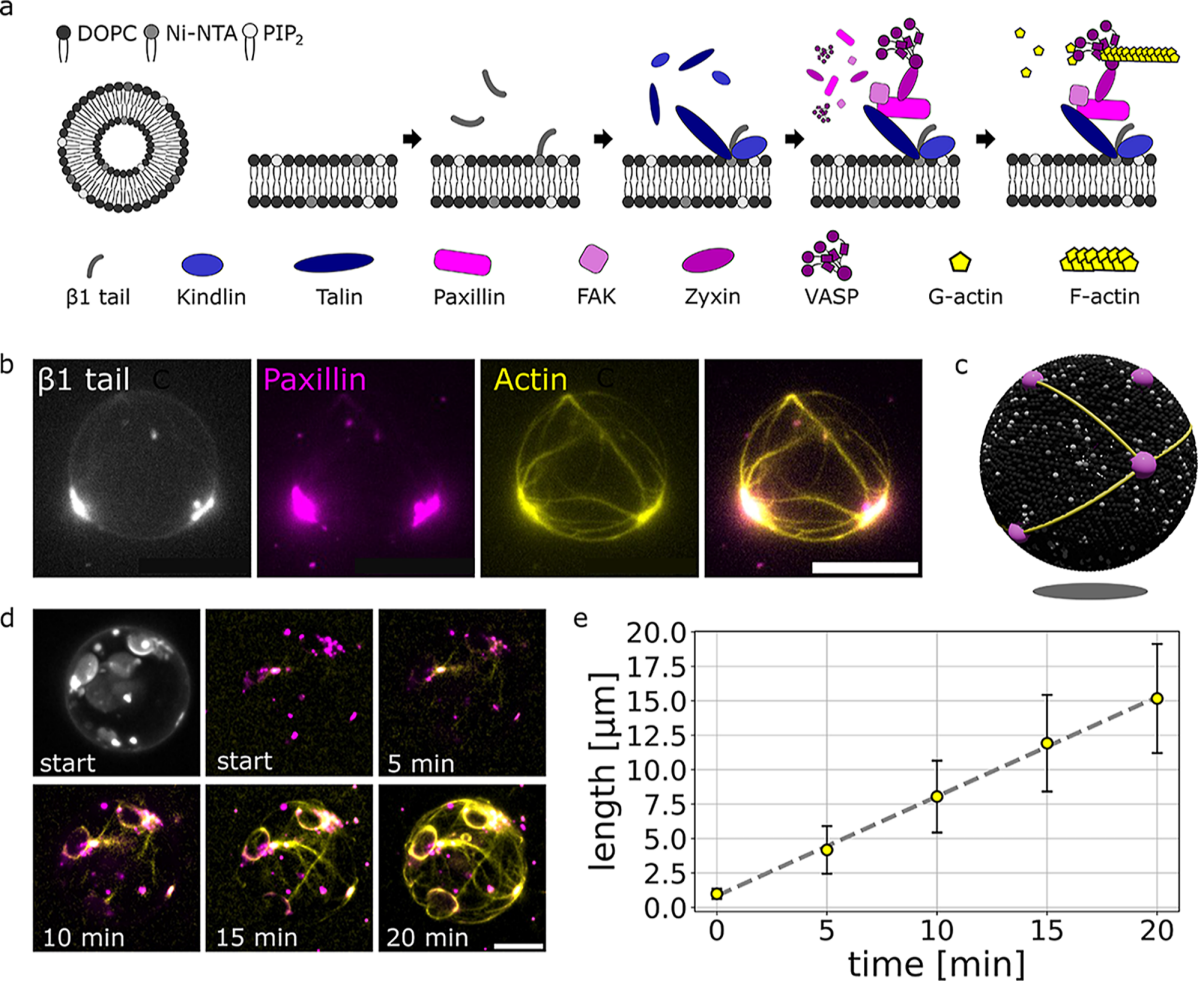
The growth of actin filaments from focal adhesion protein complexes on GUVs. Actin filaments grow from focal adhesion protein complexes on GUVs, connecting the complexes with an actin network. The complexes anchor the actin filaments toward the lipid membrane. (a) Schematic of the reconstituted model vesicles and the incubation steps driving the complex formation and allowing for actin binding and polymerization. In the last step, myosin is introduced to allow for actin network deformations. (b) Fluorescence images (z-projections) show the formation of actin filaments (yellow) on the GUV between the protein complexes (paxillin, magenta) that colocalize with β1 clusters. Scalebars are 10 μm. (c) Schematic of actin filaments connecting the complexes on the GUV. (d) Fluorescence images (z-projections) show that actin filaments (yellow) grow and elongate from the focal adhesion protein complexes (paxillin, magenta). Scalebars are 10 μm. (e) Actin filament growth from protein complexes on GUVs over time. The tracked filament lengths in each acquired frame give a polymerization rate of 0.84 μm/min. Error bars represent the standard deviations of 21 measurements.

Actin filaments elongate from these complexes, growing toward and connecting with neighboring condensates to form a continuous network spanning the GUV (Figure 3c,d and Movie S2). Tracking filament length over time yields a polymerization rate of 0.84 μm/min (Figure 3e), confirming that the complexes maintain their ability to nucleate and anchor actin filaments in 3D. The resulting actin network mirrors that observed on SLBs, providing a direct, minimal linkage between the membrane-bound protein condensates and the cytoskeletal architecture.

### Actomyosin Actively Deforms the GUVs and Demonstrates the Functionality of the Reconstituted Complexes

We next test whether the reconstituted complexes remain functional under contractile forces by adding nonmuscle myosin IIa (NMM2) after actin network formation (Materials and Methods and Figure 4a). Upon the addition of NMM2, the actin network contracts, and the GUV membrane crumples, forming folds at sites where filaments connect two or more condensates (Figure 4b, Movies S3 and S4). The extent of deformation depends on myosin concentration: higher concentrations produce greater shape changes, with circularity decreasing to ≈0.5 (Figure 4c). Without the assembly of focal adhesion-like complexes on the GUVs, the contractile force of actomyosin cannot be transmitted to the GUVs (Figure S5).

**Figure 4:**
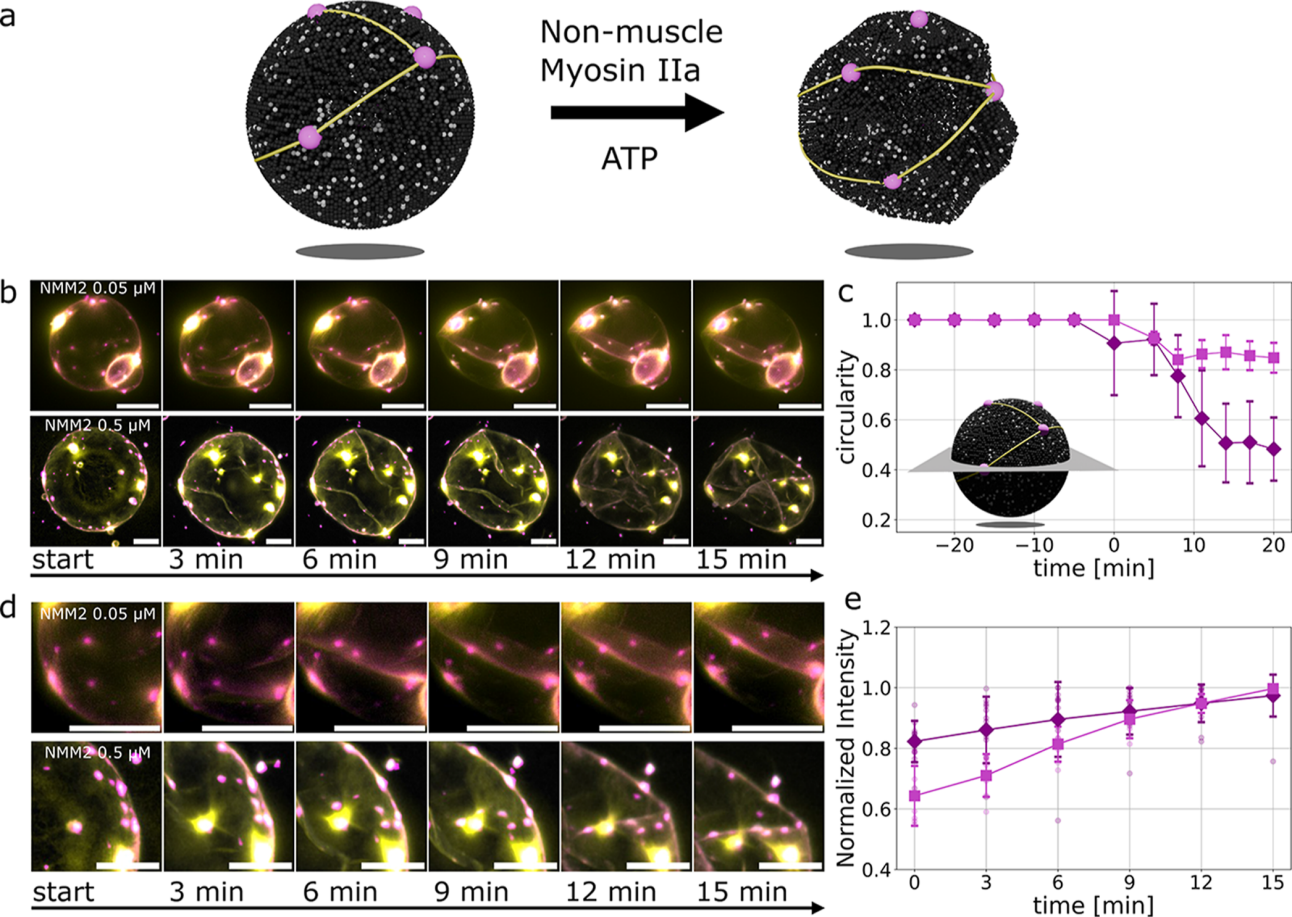
Actomyosin deforms GUVs. (a) Schematic of the addition of nonmuscle myosin IIa (NMM2) after the actin network formation on a GUV. (b) Fluorescence images (z-projections) show the GUVs’ deformation after the addition of NMM2 (0.05 μM, top; 0.5 μM, bottom). The GUVs are covered by actin filaments (yellow) that are anchored to the protein complexes (paxillin, magenta). Scale bars are 10 μm. (c) Circularities in the vesicles’ midplane as a function of time. At time point 0, NMM2 (0.5 μM, diamond; 0.05 μM, square) is added to the GUVs. Immediately after, the midplane shape is deformed and shows less circularity. Error bars represent the standard deviations of 10 measurements. (d) Fluorescence images (z-projections) of the deforming vesicles show how the anchor points (paxillin, magenta) of the actin network (yellow) around the vesicle are moved along the actin filaments and across the membrane. Scale bars are 10 μm. (e) Normalized line intensities along the actin filaments during deformation with NMM2 (0.5 μM, diamond; 0.05 μM, square) between two complexes connected to the filament, measured in the maximum projection of fluorescence images. The single data points show single filaments from different experiments. Error bars represent the standard deviations of 10 measurements.

During contraction, thin actin bundles reorganize into thicker bundles within the folds, accompanied by an increase in actin fluorescence intensity along these regions (Figure 4d,e, Movies S5 and S6). At lower myosin concentrations (0.05 μM), this bundling effect is more pronounced, while higher concentrations (0.5 μM) appear to drive simultaneous contraction of many filaments, reducing the relative bundling increase. The less bundling at higher NMM2 concentration indicates the enhanced polymerization by myosin-induced fragmentation of actin filaments, which creates new barbed ends for actin polymerization.^34^ As the actin network deforms, membrane-bound protein complexes move closer together and align along the folds, indicating that the assemblies remain stably anchored and respond dynamically to the changing membrane geometry (Figure 4d).

## Discussion

We show that minimal focal adhesion-like complexes can be reconstituted on the three-dimensional model system of giant unilamellar vesicles (GUVs) and retain their ability to recruit actin, transmit forces, and remain anchored under load. These complexes assemble directly on membranes containing PIP_2_ and integrin β1 tails, gradually decreasing the diffusivity of the β1 tails on the membrane as protein–protein and protein–lipid interactions build up. The decreasing diffusivity and mobile fraction of β1 tails reflects the condensate-like nature of the assemblies, consistent with previous reports of LLPS-driven focal adhesion formation on supported lipid bilayers (SLBs) and in cells.^30,35^

The reconstituted complexes nucleate and anchor actin filaments, forming interconnected cortical networks similar to those observed in SLB-based actin assembly systems.^36–38^ However, unlike GUV studies that relied on artificial linkers such as biotin-streptavidin^39,40^ or biotin-neutravidin,^7,9,15,16^ our approach maintains native-like coupling between the membrane, focal adhesion proteins, and actin filaments. This direct linkage allows us to probe how adhesion complexes withstand myosin-generated forces in a fully three-dimensional context.

Upon NMM2 addition, actomyosin contraction thickens filament bundles, aligns complexes, and deforms vesicles without detachment, revealing that defined protein–membrane interactions alone are sufficient for stable force transmission. Our findings bridge previous work on LLPS-mediated adhesion assembly^29,30,33^ and actin-driven shape changes in GUVs.^9,10^ Prior SLB studies have shown that actin polymerization can modulate membrane mechanics, but few have incorporated both native anchoring proteins and active force generation.^41,42^ The use of nonmuscle myosin IIa in our reconstruction system causes moderate contraction on the GUVs due to the lower force generation of NMM2 against the force-sensitive actin binding of paxillin and talin. Unlike the total collapse of the actin network seen in experiments with skeletal myosin,^7,8,39,41^ using NMM2 allows future studies to investigate the behaviors of FA proteins during actomyosin contraction. By integrating FA-like condensates with actomyosin contractility, our system captures essential mechanobiological functions–actin recruitment, force transmission, and structural stability under load–while eliminating the complexity of cellular signaling networks.

This controllable synthetic platform opens opportunities to dissect the physical principles of mechanosensing, systematically test the contribution of individual adhesion components (e.g., vinculin, α-actinin),^36,43^ and explore how protein condensates adapt to sustained mechanical stress.^44^ Beyond fundamental studies, the approach provides a blueprint for engineering force-responsive biomimetic structures with tunable mechanical properties.

## Materials and Methods

### Materials and Reagents

Isopropyl-β-d-thiogalactopyranoside (IPTG), tris, tris­(2-carboxyethyl)­phosphine (TCEP), hydrogen peroxide (H_2_O_2_), sulfuric acid (H_2_SO_4_), imidazole, sodium hydroxide (NaOH), potassium chloride (KCl), zinc chloride (ZnCl_2_) sodium azide (NaN_3_), magnesium chloride (MgCl_2_), calcium chloride (CaCl_2_), citric acid, tetrasodium diphosphate (Na_4_P_2_O_7_), acetic acid, potassium iodide (KI), ammonium sulfate ((NH_4_)_2_SO_4_), monosodium phosphate (NaH_2_PO_4_) ethylenediaminetetraacetic acid (EDTA), 3,12-bis­(carboxymethyl)-6,9-dioxa-3,12-diazatetradecane-1,14-dioic acid (EGTA), Atto 488 NHS-ester, Atto 565 NHS-ester, DMSO, dithiothreitol (DTT), glucose, pyranose-oxidase (PO), catalase (C), adenosine triphosphate (ATP), creatine phosphokinase (CPK), creatine phosphate (CPH), methyl cellulose (MC), bovine serum albumin (BSA), and phosphate buffered saline (PBS) tablet were purchased from Sigma-Aldrich. 1,2-Dioleoyl-*sn*-glycero-3-phosphocholine (DOPC), 1,2-dioleoyl-*sn*-glycero-3-[(*N*-(5-amino-1-carboxypentyl)­iminodiacetic acid)­succinyl] (nickel salt) (DGS-NTA-Ni), 1,2-dioleoyl-*sn*-glycero-3-phosphoethanolamine-*N*-[methoxy­(polyethylene glycol)-2000] (ammonium salt) (PEG2000-PE), and 1,2-dioleoyl-*sn*-glycero-3-phospho-(1′-myo-inositol-4′,5′-bisphosphate) (ammonium salt) (PIP_2_) were purchased from Avanti Polar Lipids.

### Buffers

G-actin buffer (G-buffer) (pH 8.0) contains 2 mM Tris, 0.2 mM ATP, 0.2 mM CaCl_2_, 0.2 mM DTT, and 0.8 mM NaN_3_. KMEI buffer (pH 7.4) contains 10 mM Imidazole, 100 mM KCl, 1 mM EGTA, 1 mM MgCl_2_, and 2.5 μM ZnCl_2_. Tris buffer (pH 8.0) contains 50 mM Tris and 200 mM NaCl. PBS buffer (pH 7.4) contains 10 mM phosphate buffer, 2.7 mM KCl, and 137 mM NaCl. Citric buffer (pH 4.85) contains 20 mM citric acid, 50 mM KCl, 0.1 mM EDTA, and 0.1 mM NaN_3_.

### Protein Expression, Purification and Labeling

Atto643-labeled his_6_-β1 integrin cytoplasmic tail peptides were synthesized and purified by the Max Planck Institute of Biochemistry (MPIB) Core Facility. The correct peptide sequence was controlled by high-resolution intact mass spectrometry.

Kindlin-2 and paxillin were expressed and purified as described earlier.^45^ Briefly, an N-terminal his-sumo tag was added to express the proteins soluble in *Escherichia coli* (DE3) Rosetta 2. After cell lysis, the proteins were captured with a Ni-NTA-immobilized metal ion affinity chromatography (IMAC); the sumo tag was removed by SenP2 digest and subsequent pull-down with Ni-NTA beads, followed by a final size exclusion chromatography.

His-tagged talin-1 was expressed and purified as described^46^ with minor changes. The protein was expressed in *E. coli* (DE3) Rosetta 2 upon induction with 0.2 mM IPTG overnight at 18 °C. The cells were harvested by centrifugation and lysed by sonication in IMAC running buffer (25 mM Tris (pH 7.8), 500 mM NaCl, and 1 mM TCEP) supplemented with 2 mM MgCl_2_, 40 μL DNaseI (obtained from MPIB Core Facility), and a spatula tip of lysozyme. After clearing the lysate, the sample was loaded on a 5 mL HisTrap HP Ni-NTA IMAC column (17524801; Cytiva), washed with IMAC running buffer, and eluted with a stepwise gradient with IMAC elution buffer (25 mM Tris (pH 7.8), 500 mM NaCl, 1 mM TCEP, and 500 mM imidazole). Elution fractions were pooled, concentrated, and further purified with a Superose 6 Increase 10/300 GL size exclusion chromatography (29091596, Cytiva) using 20 mM Tris, pH 7.8, 200 mM NaCl, and 1 mM TCEP as running buffer.

Atto 565-NHS-ester was dissolved in DMSO at a concentration of 5 μg/μL. Kindlin-2, talin-1, or paxillin was rebuffered to PBS buffer using Zeba Spin Desalting Columns (7K, 0.5 mL; Thermo Scientific) and mixed with the Atto 565-NHS-ester solution in a mole ratio of 1:2.5. The mixture was kept for 2 h at room temperature. After the 2 h reaction, the mixture was rebuffered to Tris Buffer to quench the reaction and remove the excess dye. The labeled proteins were stored at −80 °C.

Murine focal adhesion kinase carrying an N-terminal his-sumo tag was cloned using Gateway cloning into PB-T-Rfa to establish a stable FAK-expressing HEK293T cell line.^47^ The cells were cultured in FreeStyle 293 Expression Medium (12338018, Gibco) until reaching a cell density of 10^6^ cells per mL before inducing protein expression by doxycycline addition (1 μg/mL) for 3 days. Cells were collected by centrifugation, resuspended in IMAC running buffer (25 mM Tris (pH 7.5), 500 mM NaCl, 10% glycerol, and 1 mM TCEP) supplemented with a cOmplete, EDTA-free Protease Inhibitor tablet and 40 μL Benzonase (obtained from MPIB Core Facility) and lysed by douncing on ice. Centrifugation-cleared lysate (60 min, 58,000*g*, 4 °C) was sterile-filtered, applied to Ni-NTA IMAC column (HisTrap SP, 5 mL, Cytiva), washed with IMAC running buffer and eluted with a stepwise gradient with IMAC elution buffer (25 mM Tris (pH 7.5), 500 mM Imidazole, 500 mM NaCl, 10% glycerol, and 1 mM TCEP). The protein was diafiltered using a 30 kDa cutoff Amicon Ultra 15 (UFC903024; Merck Millipore) filter against IMAC running buffer, and the his-sumo-tag was cleaved using sumo protease (obtained from MPIB Core Facility) overnight at 4 °C. The cleaved protein was further purified with size exclusion chromatography to remove any protein aggregates using a Superdex 200 Increase 10/300 GL column (28-9909-44; Cytiva) in phosphate-buffered saline supplemented with additional 150 mM NaCl and 1 mM TCEP.

Human his-tagged zyxin-mcherry was expressed in *E. coli* (DE3) BL21 upon induction with 0.2 mM IPTG overnight at 18 °C. One h before induction, the media was supplemented with 0.2 mM Betain-hydrochlorid and 100 μM ZnCl_2_. Cells were harvested by centrifugation and lysed by sonication in IMAC running buffer (50 mM Tris (pH 7.5), 300 mM NaCl, 20 mM Imidazole, 10% glycerol, 0.05% Tween 20, 0.5 mM EDTA and 1 mM DTT) supplemented with mM MgCl_2_, 2 U/mL Benzonase, 40 μg/mL lysozyme and one tablet of cOmplete, EDTA-free Protease Inhibitor Cocktail. After clearing the lysate, the sample was loaded to 3 mL of cOmplete His-tag Purification Resin, washed with IMAC running buffer, running buffer containing 500 mM NaCl and 2 mM ATP, and eluted with IMAC elution buffer (50 mM Tris-HCl (pH 7.5), 300 mM NaCl, 10% glycerol, 500 mM Imidazole and 1 mM DTT). Protein was loaded to the 5 mL Hitrap Q HP column and eluted with a gradient of 0–1 M NaCl, 30 mM Tris (pH 7.5), 0.5 mM EDTA, 10% glycerol, 1 mM DTT. Elution fractions were concentrated and his-tag was cleaved with TEV-Protease overnight at 10 °C. After cleavage 20 mM Imidazole was added to the protein sample and protein applied to 5 mL HisTrap HP Ni-NTA IMAC column, washed with IMAC running buffer, and eluted with IMAC elution buffer (50 mM Tris (pH 7.5), 300 mM NaCl, 20 mM Imidazole, 10% glycerol, 0.5 mM EDTA and 1 mM DTT). Unbound protein was collected and further purified with HiLoad 16/600 Superdex 200 pg size exclusion chromatography column coupled to HiTrap Q 1 mL column in front using IEX elution buffer (50 mM HEPES (pH 7.5), 200 mM NaCl, 10 μM ZnCl_2_, 5% glycerol and 1 mM DTT) as running buffer. All purification steps were done at 4 °C.

Human His-tagged VASP containing the high-affinity GAB of *Dictyostelium discoideum* VASP was expressed and purified as described.^37^ Briefly, protein was expressed in *E. coli* (DE3) BL21. After cell lysis, the proteins were captured with a Ni-NTA-immobilized metal ion affinity chromatography (IMAC), followed by a final size exclusion chromatography. The protein was then concentrated, frozen in liquid nitrogen, and stored at −80 °C.

Rabbit skeletal muscle actin was purified from acetone powder.^48^ No rabbits were directly involved in this study. Monomeric actin (G-actin) was stored in G-buffer at 4 °C. For fluorescent actin, surface-exposed lysines were labeled with Atto 488 NHS-ester.

Nonmuscle myosin IIa (NMM2) was extracted and purified from human thrombocytes with an adapted protocol.^49^ Expired thrombocyte donations were gifted by Klinikum Rechts der Isar. The thrombocytes were centrifuged (10 min, 800*g*, room temperature), resuspended in 100 mL of PBS buffer, and centrifuged (10 min, 800*g*, room temperature) again. The pellet was then resuspended in extraction buffer (30 mM imidazole (pH 7.0), 900 mM KCl, 15 mM Na_4_P_2_O_7_, 5 mM MgCl_2_, and 3 mM DTT) at a concentration of 0.5 mg/mL while stirring on ice for 30 min. The solution was filtered and then mixed with an ice-cold 2 mM MgCl_2_ solution at a volume ratio of 1:3 while stirring. The pH of this solution was then adjusted to pH 6.4 using 0.5 M acetic acid and stirring on ice for 15 min. The actomyosin is then collected by centrifugation (30 min, 39,800*g*, 4 °C). The resulting actomyosin pellet is resuspended in 10 mL of KI-ATP buffer (20 mM imidazole (pH 7.0), 600 mM KI, 5 mM ATP, 5 mM DTT, and 1 mM MgCl_2_) using a Dounce homogenizer to depolymerize the F-actin. The solution was then centrifuged (30 min, 39,800*g*, 4 °C). From the supernatant, the NMM2 was precipitated using (NH_4_)_2_SO_4_ fractionation. For that, an ice-cold saturated (NH_4_)_2_SO_4_ solution (3.93 M) with 10 mM EDTA (pH 7.0) was added. The first step was done by slowly adding 0.36 mL per ml of protein solution (to a concentration of 1.04 M), stirring for 10 min on ice, and centrifuging (15 min, 39,800*g*, 4 °C). Next, 0.478 mL of (NH_4_)_2_SO_4_ solution per ml of supernatant (to a concentration of 1.97 M) was slowly added, stirred for 10 min on ice, and centrifuged (15 min, 39,800*g*, 4 °C). This pellet containing the NMM2 proteins was resuspended in 8 mL of KI-ATP buffer, centrifuged (15 min, 39,800*g*, 4 °C), and applied to a HiLoad 26/600 Superdex 200 size-exclusion column (28-9893-36, Cytiva) equilibrated with KI-ATP buffer. NMM2 was precipitated by adding 2× volume equivalents of cold saturated (NH_4_)_2_SO_4_ solution and centrifuged (15 min, 39,800*g*, 4 °C). The pellet was dissolved in 1 mL of myosin buffer (10 mM NaH_2_PO_4_, pH 7.0, 600 mM KCl, and 60% (w/vol) sucrose) and stored at −80 °C.

Mouse profilin 2a was expressed as a glutathione *S*-transferase (GST) fusion protein and purified as described in.^37^ Briefly, protein was expressed in *E. coli* (DE3) BL21. After cell lysis, the proteins were captured with a glutathione sepharose, the GST tag was cleaved, and the eluted protein was then dialyzed overnight at 4 °C against 20 mM Tris, pH 7.0, 150 mM NaCl, 1 mM DTT, flash-frozen in liquid nitrogen, and stored at −80 °C.

Capping protein expressed as a heterodimer of mouse α1- and human β2 subunit cloned into pRFSDuet-1 (71341, Novagen) as described in.^37^ Protein was expressed in *E. coli* (DE3) BL21 and the expression was induced with 0.5 mM IPTG and incubation at 26 °C overnight. Bacteria were harvested and lysed by French pressing in a 20 mM Tris, pH 8.0, 10 mM imidazole, 250 mM NaCl, 1 mM EDTA, 1 mM DTT, 0.5 tablet of cOmplete protease inhibitor, 5% glycerol, and 2 U/mL Benzonase buffer. After centrifugation, the supernatant was incubated with Anti-FLAG M2 affinity gel (A2220, Sigma) at 4 °C for 90 min. After washing, 140 μL FLAG peptide (25 mg lyophilized powder per 1.4 mL buffer) were added and incubated at 4 °C for 1 h. The beads were spun down and the supernatant was dialyzed overnight at 4 °C against 10 mM Tris, pH 8.0, 50 mM KCl, and 1 mM DTT. The protein was then frozen in liquid nitrogen and stored at −80 °C.

### Reconstitution on Supported Lipid Bilayers

#### Small Unilamellar Vesicle Production

Lipid mixtures (87.5 mol % DOPC, 5 mol % DGS-NTA-Ni, 5 mol % PIP_2_, and 2.5 mol % PEG2000-PE) were mixed in chloroform. Lipid films were prepared by drying the lipid mixture under a stream of nitrogen and placed under a vacuum for at least 2 h. Lipid films were hydrated and resuspended by sonication for 30 min in Citric buffer at a final lipid concentration of 0.5 mM. Small unilamellar vesicles (SUVs) were then created by extruding lipid suspensions 20 times through 100-nm-pore membrane filters (Whatman) using the mini-extruder (Avanti Polar Lipids). SUVs were stored at 4 °C and used within 72 h.

#### Flow Chamber Preparation

Flow chambers (≈40 μL) that consist of coverslips (22 mM × 22 mM; Carl Roth) fixed to microscope slides (25 mM × 75 mM; Carl Roth) by 3-layer parafilm were used for the assays. The coverslips were sonicated for 30 min in 3 M NaOH and rinsed with Milli-Q H_2_O before being cleaned in piranha solution (2:1, H_2_SO_4_/H_2_O_2_) for 10 min to render the surface hydrophilic. The piranha-cleaned coverslips were then rinsed and stored in Milli-Q H_2_O. The microscope slides were sonicated for 30 min in 2 wt % Hellmanex aqueous solution (Hellma) and rinsed with Milli-Q H_2_O before being stored in ethanol. The coverslips and microscope slides were used within 72 h after cleaning.

#### 2D Model Membrane System Reconstitution

Supported lipid bilayers (SLBs) were prepared in flow chambers. SUVs were added to chambers at a final lipid concentration of 0.167 mM and allowed to rupture and form an SLB on the glass surface for 20 min at room temperature. Afterward, the SLBs were first washed with 0.8 mL citric buffer,^50^ and then 0.8 mL KMEI buffer to remove excess liposomes. SLBs were incubated with 0.5 μM his_6_-tagged integrin β1 tails in KMEI buffer for 20 min and then washed with 0.8 mL KMEI buffer. The β1-bound SLBs were incubated with 0.5 μM kindlin-2 and 0.5 μM talin-1 in KMEI buffer for 30 min; then with 0.2 μM paxillin (10% labeled), 0.1 μM FAK, 0.2 μM zyxin, and 0.2 μM VASP in KMEI buffer for 30 min. The polymerization mixture was introduced after the incubation of focal adhesion proteins. The polymerization mixture contains 2 μM G-actin (12.5% labeled), 0.05 μM capping protein, 10 μM profilin, 1 mM ATP, 1 mM DTT, 8 U/mL PO, 1.7 kU/mL C, and 36 mM glucose in KMEI buffer. In this mixture, PO and C function as an oxygen-scavenging system to prevent protein denaturation and photobleaching during fluorescence imaging.

### Reconstitution on Giant Unilamellar Vesicles

#### Electroformation

Giant unilamellar vesicles (GUVs) were produced using electroswelling (Vesicle Prep Pro; Nanoion). Lipid mixture (87.5 mol % DOPC, 5 mol % DGS-NTA-Ni, 5 mol % PIP_2_, and 2.5 mol % PEG2000-PE) was mixed in chloroform. Eleven μL of 5 μM lipid mixture was dried on indium tin oxide coated glass slides under a vacuum for at least 2 h. The dried lipid films were then rehydrated with 300 μL of inner solution (200 mM sucrose, 3.3 mM imidazole, 33 mM KCl, 0.33 mM EGTA, and 0.33 mM MgCl_2_). The osmolality of the inner solution was prepared to be 5–10 mOsm/kg lower than the outer buffer with proteins in the following incubation steps. The electroformation protocol applied a voltage up to 3 V and a frequency of 750 Hz. After electroformation, the GUVs were transferred into 6 mL of KMEI buffer.

#### 3D Model Membrane System Reconstitution

The reconstruction on GUVs was performed in well chambers (sticky-slide 8 Well; ibidi), where each chamber was passivated with a BSA solution (10 mg/mL) for 20 min before adding 200 μL of the GUVs in KMEI buffer.

For the protein incubation, in each step, 150 μL of the buffer was removed from the chamber, and 150 μL of the protein mixture was added to reach the final protein concentrations. 0.5 μM his_6_-tagged integrin β1 tails in KMEI buffer for 20 min. The β1-bound GUVs were incubated with 0.5 μM kindlin-2 and 0.5 μM talin-1 in KMEI buffer for 30 min; then with 0.2 μM paxillin (10% labeled), 0.1 μM FAK, 0.2 μM zyxin, and 0.2 μM VASP in KMEI buffer for 30 min. The polymerization mixture was introduced after the incubation of focal adhesion proteins. The polymerization mixture contains 2 μM G-actin (12.5% labeled), 0.05 μM capping protein, 10 μM profilin, 1 mM ATP, 1 mM DTT, 8 U/mL PO, 1.7 kU/mL C, and 36 mM glucose in KMEI buffer. The NMM2 mixture was introduced after the formation of the actin network on the GUVs. The NMM2 mixture contains 0.05/0.5 μM NMM2, 0.4 wt % MC, 1 mM ATP, 18 U/mL CPK, 9 mM CPH, 8 U/mL PO, 1.7 kU/mL C, and 36 mM glucose. In this mixture, PO and C function as an oxygen-scavenging system to prevent protein denaturation and photobleaching during fluorescence imaging. CPK and CPH serve as an ATP-regeneration system.

### Imaging and Data Acquisition

A Leica DMi8 microscope with an HC PL APO 100×/1.47 oil immersion objective was used to perform the epifluorescence and total internal reflection fluorescence imaging for SLBs using an ORCA-Flash 4.0 CMOS camera (C13440-20CU; Hamamatsu, Shizuoka, Japan). A Leica Infinity Scanner unit was used to perform fluorescence recovery after photobleaching (FRAP) experiments. A Leica Thunder Imaging System with an HCX PL APO 63×/1.40 oil immersion objective was used to image the GUVs. Fiji/ImageJ was used to analyze the filament length and GUV centricity.

### FRAP Experiemnt

A circle with a diameter of 5 μm was bleached with a 638 nm laser, and fluorescence images were acquired for 80 s. A region of the SLB outside the circle was used for background subtraction. Fluorescence intensity values within bleached regions were exported using LAS X software. Data were normalized to prebleach levels and fitted to a double-component exponential recovery function, $F\left(t\right)=y_{0}+A_{fast}\cdot e^{-t/\tau_{fast}}+A_{slow}\cdot e^{-t/\tau_{slow}}$ , where *F*(*t*) is the relative fluorescence intensity over time, $\frac{-\left(A_{fast}+A_{slow}\right)}{1-\left(y_{0}+A_{fast}+A_{slow}\right)}$ is the mobile fraction, τ_fast_ is the fast recovery time constant, and τ_slow_ is the slow recovery time constant. Fitting was performed using Python 3.

## Supplementary Material















## Notes

Movies S1 (AVI)
Movies S2 (AVI)
Movies S3 (AVI)
Movies S4 (AVI)
Movies S5 (AVI)
Movies S6 (AVI)
Additional experimental details, materials, and methods for the visualization of PIP_2_ in the GUVs, visualization of kindlin-2 and talin-1 on the GUVs, and dye influx assay for membrane permeability (PDF)
