# Supplementary material for: Force Transmission by Minimal Focal Adhesion Complexes Induces Synthetic Cell Deformation
Source: ACS Synth Biol. 2025 Dec 17;15(1):223–32. doi: 10.1021/acssynbio.5c00645 (PMC12814769; doi:10.1021/acssynbio.5c00645)
Supplement: Supplementary file 7 [file sb5c00645_si_007.pdf]

Supporting Information for Force Transmission by Minimal Focal Adhesion Complexes Induces Synthetic Cell Deformation

Natalie Huhn,[†,‡,§] Chiao-Peng Hsu,[*,†] Timon Nast-Kolb,[†] Arsenii Hordeichyk,[†,¶] and Andreas R. Bausch[*,†,¶]

†*Heinz Nixdorf Chair in Biophysical Engineering of Living Matter, Center for Functional Protein Assemblies, Center for Organoid Systems, Department of Bioscience, Technical University of Munich, Technical University of Munich School of Natural Sciences, Garching 85748, Germany*

‡*Department of Physics, Friedrich-Alexander-Universität Erlangen-Nürnberg, Erlangen 91058, Germany*

¶*Max Planck School Matter to Life, Jahnstraße 29, D-69120 Heidelberg, Germany*

§*Clinic for Children and Adolescent Medicine, Friedrich Alexander University Erlangen-Nuremberg, Erlangen 91054, Germany*

E-mail: chiao-peng.hsu@tum.de; abausch@mytum.de

Visualization of PIP_2 in the GUVs

1-oleoyl-2-(6-((4,4-difluoro-1,3-dimethyl-5-(4-methoxyphenyl)-4-bora-3a,4a-diaza-s-indacene-2-propionyl)amino)hexanoyl)-sn-glycero-3-phosphoinositol-4.5-bisphosphate (ammonium salt) (TMR PIP_2) was purchased from Avanti Polar Lipids.

Lipid mixture with labeled PIP_2 (87.5 mol% DOPC, 5 mol% DGS-NTA-Ni, 1 mol% PIP_2, 4 mol% TMR PIP_2, and 2.5 mol% PEG2000-PE) was used to produce GUVs using electroswelling as described in the manuscript.

Visualization of kindlin-2 and talin-1 on the GUVs

The assembly of 0.5 µM kindlin and 0.5 µM talin β1-bound PIP_2-containing GUVs was performed as described in the manuscript. 10 mol% labeled kindlin-2 or talin-1 was used to visualize the proteins on the membranes (Figure S3).

Dye influx assay for membrane permeability

Sulforhodamine B (Sigma-Aldrich) was used to visualize dye influx across the GUVs. The GUVs without protein incubation and the GUVs with stepwise protein incubation, as described in the manuscript (0.5 µM β1, 0.5 µM kindlin-2, 0.5 µM talin-1, 0.2 µM paxillin, 0.1 µM FAK, 0.1 µM zyxin, and 0.2 µM VASP), were added in KMEI buffer with 5 µM sulforhodamine B. A Leica TCS SP5 confocal microscope with an HCX PL APO 63×/1.40 CS2 oil immersion objective was used to image the vesicles. The normalized intensity is defined as the dye intensity inside and outside a GUV, I_{in}/I_{out}.

Movie S1:

The polymerization of actin filaments from protein complexes on a SLB. Images are taken every 20 s. β1-tail is depicted in greyscale, paxillin in magenta, and actin in yellow.

Movie S2:

The polymerization of actin filaments from protein complexes on a GUV. Images are taken every 5 min. β1-tail is depicted in greyscale, paxillin in magenta, and actin in yellow.

Movie S3:

The deformation of a GUV with a myosin concentration of 0.05 µM. Images are taken every 3 min. β1-tail is depicted in greyscale, paxillin in magenta, and actin in yellow.

Movie S4:

The deformation of a GUV with a myosin concentration of 0.5 µM. Images are taken every 3 min. β1-tail is depicted in greyscale, paxillin in magenta, and actin in yellow.

Movie S5:

Actin bundling and complex movement during the deformation of a GUV with a myosin concentration of 0.05 µM. Images are taken every 3 min. β1-tail is depicted in greyscale, paxillin in magenta, and actin in yellow.

Movie S6:

Actin bundling and complex movement during the deformation of a GUV with a myosin concentration of 0.5 µM. Images are taken every 3 min. β1-tail is depicted in greyscale, paxillin in magenta, and actin in yellow.

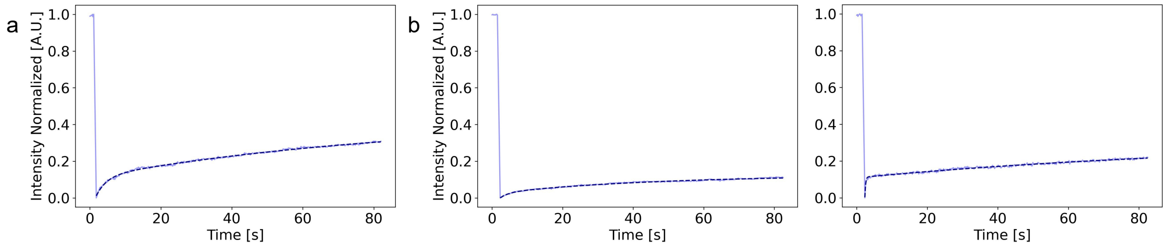

Figure S1: **FRAP recovery curves of paxillin and actin.** (a) FRAP recovery curve of the labeled paxillin after 30 min incubation of 0.2 µM paxillin (10 % labeled), 0.1 µM FAK, 0.2 µM zyxin, and 0.2 µM VASP. (b) FRAP recovery curves of paxillin (left) and actin (right) after the addition of actin polymerization mixture. The recovery of paxillin decreases with the assembly of the actin filaments. Dotted curves show the double-component exponential recovery fittings.

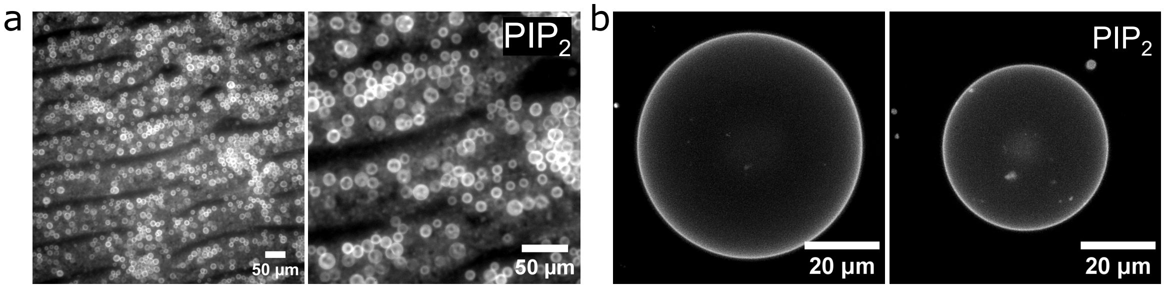

Figure S2: **Fluorescence microscopy images of GUVs showing the fluorescence intensity of PIP$_2$.** (a) Fluorescence images taken during the electroswelling process show the incorporation of PIP$_2$ into the GUVs' membranes. (b) PIP$_2$ stays homogeneously distributed across the vesicles' membranes one hour after swelling.

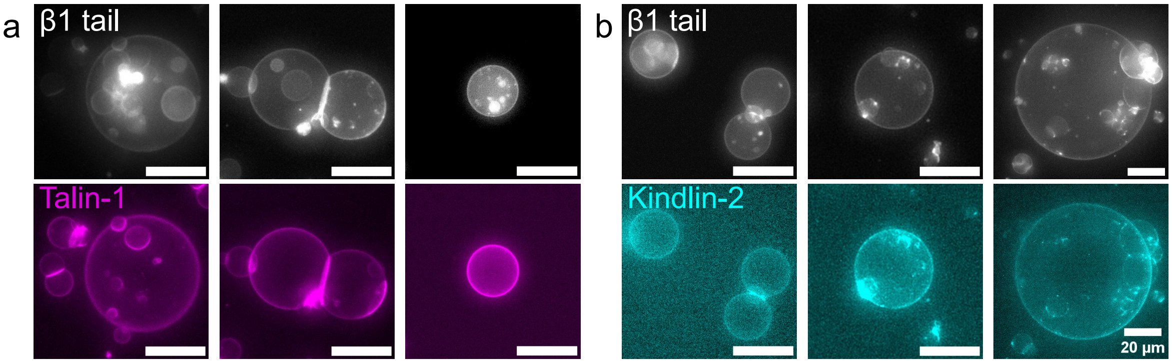

Figure S3: **Fluorescence microscopy images of GUVs with talin-1 and kindlin-2 binding to the β1 tail functionalized GUV membrane.** After incubating 0.5 µM of talin-1 (a) and kindlin-2 (b) onto the PIP$_2$-containing membrane for 30 min, the vesicles' membranes show high intensity in the labeled protein channel.

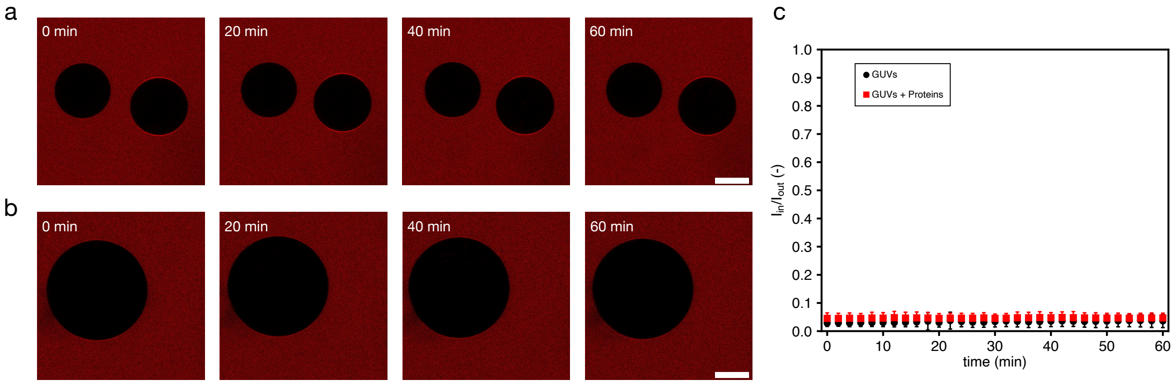

Figure S4: **Membrane permeability is not influenced by the assembly of protein complexes.** Confocal images (mid-plane) of (a) GUVs and (b) GUVs with stepwise protein incubation (0.5 µM β1, 0.5 µM kindlin-2, 0.5 µM talin-1, 0.2 µM paxillin, 0.1 µM FAK, 0.1 µM zyxin, and 0.2 µM VASP). Scale bars are 20 µm. (c) Normalized dye intensity inside and outside a GUV (I_{in}/I_{out}) in either case. Error bars represent the standard deviations of 45 (GUVs) and 25 (GUVs with proteins) measurements.

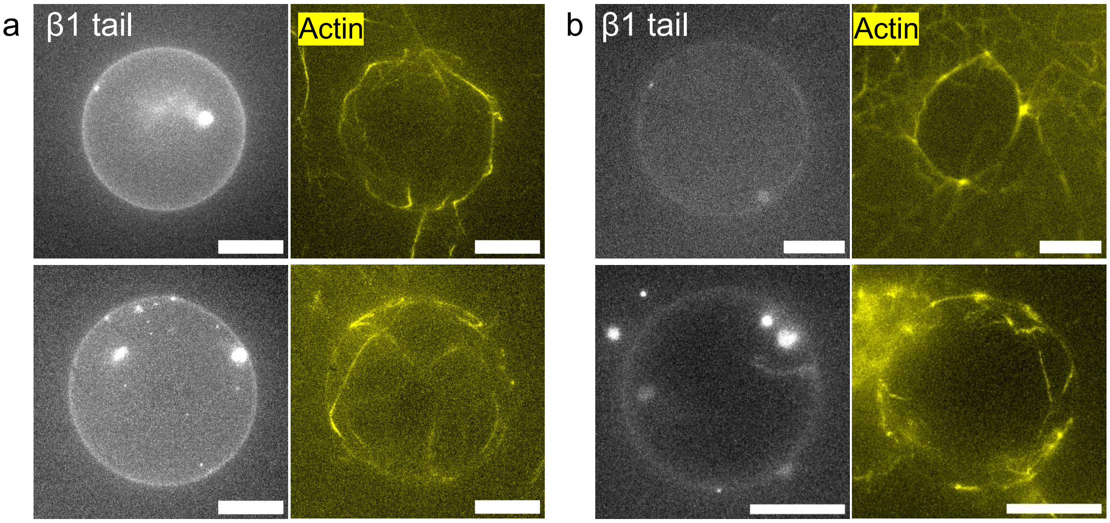

Figure S5: **No contractile force is transmitted without the assembly of focal adhesion–like complexes on the GUVs.** Fluorescent images (z-projections) of the GUVs with integrin β1 tail protein (greyscale) on their surface with actin (yellow) bundled by VASP. (a) GUVs covered by a network of polymerized actin without anchoring towards the membrane in the myosin mixture without NMM2. (b) Fluorescent images (z-projections) of the GUVs after the addition of NMM2. Actin network contraction either squeezes the GUV from the bottom (upper images) or the actin network collapses around the GUV after fully contracting (lower images). Scale bars are 10 µm.
